# PROTOCOL: The association between adverse childhood experiences and employment outcomes: Protocol for a systematic review

**DOI:** 10.1002/cl2.70002

**Published:** 2024-10-31

**Authors:** Amarech Obse, Evdoxia Gkaintatzi, Paul McCrone

**Affiliations:** ^1^ The Institute for Lifecourse Development University of Greenwich London UK

**Keywords:** adverse childhood experiences, employment, life course

## Abstract

There is growing evidence of a link between adverse childhood experiences (ACEs) and health and economic outcomes. Previous systematic reviews synthesised evidence of the relationships between ACEs and various health and some economic outcomes such as healthcare costs and educational attainment. The primary aim of this systematic review is to synthesise the evidence on the relationship between ACEs and employment outcomes. MEDLINE, Psychology and Behavioural Sciences Collection (APA PsycInfo), ECONLIT, Sociological Abstracts, Social Science Research Network (SSRN) and Scopus will be searched using a predefined search strategy. Cross‐sectional, cohort, or longitudinal studies published between 2000 and 2024 will be included. ACEs include abuse, neglect, household dysfunction, bullying, foster care, and racism that occur during childhood or adolescence. Employment outcomes include employment status, occupation, and income. Risk of bias of individual studies will be assessed using appropriate NHLBI‐NHI quality assessment tools for each type of study. Proportions or means will be used to analyse and compare outcomes. If data allows, we will conduct meta‐analysis. Sub‐group analyses (e.g., by gender, age group, type and number of ACEs, and intersections of identities of study subjects will be conducted. Further analysis will be conducted to assess the mediators of the effect of ACEs on employment outcomes. By sythesising evidence of the association between ACEs and economic wellbeing later in life, this review will add evidence to the broader literature on poverty. The results of this synthesis will inform policies on child welfare and employment. Results of the review will be published in a peer‐reviewed journal.

## 1 BACKGROUND

### 1.1 The problem, condition or issue

Adverse childhood experiences (ACEs) are potentially traumatic events that occur during childhood and/or adolescence (Boullier & Blair, 2018; Felitti et al., 1998). The original study that developed the concept of ACEs broadly grouped ACEs into three types with 10 categories: abuse (emotional, physical, sexual), neglect (emotional, physical), and household dysfunction (mental illness in the household, domestic violence, divorce/separation of parents, incarcerated parent/relative, and substance abuse by household members) (Felitti et al., 1998). While these ACEs measure adversity experienced in the home others suggested expansion of this concept to include adversity experienced at the community level, such as experiencing or witnessing foster care, racism, bullying, community violence, and living in an unsafe neighbourhood (Cronholm et al., 2015; Wade et al., 2014).

The negative impact of ACEs on health has been widely documented since the seminal paper by Felitti et al. (1998) that showed the link between ACEs and morbidity, mortality, and health risk behaviours. This is supported by neurobiological studies that assert disturbance in brain development in children exposed to early life stress, which remains evident in adulthood (Anda et al., 2010; Danese & McEwen, 2012; Soares et al., 2021). Systematic reviews and meta‐analyses have also collated evidence of the association between ACEs and several physical (e.g., cardiovascular diseases, gastrointestinal diseases, ischaemic heart diseases, somatic pain) and mental health outcomes (e.g., anxiety, depression, internalising disorder, posttraumatic stress disorder [PTSD]), behaviour problems (e.g., smoking, illicit drug use), suicidal ideation, and premature mortality (Hughes et al., 2017; Kalmakis & Chandler, 2015; Petruccelli et al., 2019). Furthermore, health outcomes related to ACEs vary depending on the forms of ACEs (Kalmakis & Chandler, 2015; Nurius et al., 2012), timing of ACEs (Hawes et al., 2021; Schalinski et al., 2016) and the intensity of exposure (Hawes et al., 2021). Progressive increase in disease burden or severity with an increasing number of adverse childhood events has also been reported (Afifi et al., 2008; Hughes et al., 2017; Petruccelli et al., 2019).

Not surprisingly, a greater burden of disease among individuals with ACEs translates to increased healthcare utilisation, which results in an increased economic burden throughout one's life course. For instance, a study in California in children 4–5 years showed 43% more emergency department visits in children with ACEs compared to those without while outpatient visits were almost double that of children without reported ACEs (Lewis et al., 2023). Another study among adults in California found 434,000 disability‐adjusted life years (DALYs) lost in 61% of study participants with ACEs which was equivalent to $102 billion dollars when DALYs were monetised with a value of $235,855 (2017 US dollars) per DALY (Miller et al., 2020).

The adverse outcomes of ACEs extend to other aspects of individuals' wellbeing such as education and employment. Children and adolescents with a history of ACEs are reported to have more non‐engagement in school, lower grades, school absenteeism, and suspension with increased intensity of adverse education outcomes with higher number of ACES (4+), after adjusting for sociodemographic variables (Crouch et al., 2019; Hardcastle et al., 2018; Houtepen et al., 2020). Furthermore, Hardcastle et al. (2018) showed that, a significantly higher odds of not getting formal qualification in participants with four or more ACEs, out of which 62% were unemployed. Other studies have also showed that adults with history of ACEs, compared to those without, are more likely to be unemployed, depend on welfare benefits, or live below the poverty line (Liu et al., 2013; Metzler et al., 2017).

### 1.2 Why is it important to do this review

Decades of research show that ACEs are significantly related to individuals’ health and wellbeing. But, the impact of ACEs extends beyond health, which has been the focus of the literature on ACEs, with small but growing evidence of the impact of ACEs on social and economic outcomes. A growing body of literature shows increased economic burden, and suboptimal educational and economic outcomes of childhood adversity (Crouch et al., 2019; Hardcastle et al., 2018; Houtepen et al., 2020). This indicates that the impact of ACEs passes across generations beyond one's own life course (Metzler et al., 2017). There is a need to synthesise the evidence of the association between ACEs and economic outcomes to provide an understanding of the broader impact of ACEs on people's welfare. We hypothesise that adults that were exposed to adversity, either at home or community level, in childhood or adolescents have suboptimal employment outcomes compared to adults not exposed to ACEs. While there are a wide range of childhood adversity that warrant investigation, given the resources for the review, this review is limited to assessing the association between employment outcomes and ACEs experienced at home (Felitti et al., 1998) and a selection of ACEs experienced outside home (racism, bullying, community violence, living in an unsafe neighbourhood and foster care) (Cronholm et al., 2015; Wade et al., 2014).

This systematic review will provide a comprehensive overview and synthesis of the available evidence of the association between ACEs on employment outcomes, pathways of impact of ACEs on employment outcomes, methodological issues, and research gaps. To our knowledge, this is the first systematic review of the correlation between ACEs at home or community level and employment outcomes.

## 2 OBJECTIVES

The primary objective of the review is:

- To determine the association between adversity in childhood and employment outcomes (status of employment, occupation, earnings) during adulthood.

The secondary objectives of the review are:

- To determine the association between childhood adversity and welfare benefit dependency, absenteeism, and/or presenteeism during adulthood.
- To describe the mechanisms or pathways which produce the relationship between ACEs and economic outcomes.
- To examine methods used to measure ACEs and assess the effects of ACEs on employment outcomes.

## 3 METHODS

### 3.1 Criteria for considering studies for this review

#### 3.1.1 Types of studies

We have developed a PECO (population, exposure, control and outcome) framework to develop the search criteria as follows (Morgan et al., 2018):

- Population: adult working age general population of age 19–64.
- Exposure: ACEs before the age of 18.
- Control: adult working age population not exposed to ACEs.
- Outcome: employment outcomes (including salary, income, or occupation).

The studies that will be included in the review will be original studies (observational, cross‐sectional, longitudinal) that examine the association between ACEs and employment outcomes. We do not have restrictions on the type of study design. However, we will exclude reports, clinical trials, books, protocols, commentary, editorial, and letters. To be included in the review, studies must report at least one employment outcome and measure the association between the employment outcome and ACEs. Employment outcomes can be described as a categorical (employed or not employed; blue‐collar or white‐collar occupation) or quantitative dependent variable (income from employment). Categorical employment outcomes can be described by odds ratio or percentages while mean or median can be used for quantitative outcomes such as income (salary or wage).

Primary independent variable of interest is ACEs experienced in the home and/or community. ACEs at home include abuse (emotional, physical, sexual), neglect (emotional, physical), and household dysfunction (mental illness in the household, domestic violence, divorce/separation of parents, incarcerated parent/relative, and substance abuse by household members) (Anda et al., 2010; Boullier & Blair, 2018; Felitti et al., 1998). Community‐level ACEs include racism, foster care, community violence, bullying, and living in an unsafe neighbourhood (Cronholm et al., 2015; Wade et al., 2014). To be included in the review, studies must include variable(s) that measure ACEs as one of the predictors for employment outcomes. ACEs are measured as categorical variables indicating whether a study participant has experienced ACEs as a child or the number and types of ACEs.

### 3.2 Types of participants

Population of interest for the review are adults (aged 18+) who were recruited from the general population in the original studies for assessing the association between ACEs and employment outcomes. Studies that focus on general description of employment outcomes in a population with history of ACEs without analysis of difference with those without a history in ACEs will be excluded.

### 3.3 Types of outcome measures

#### 3.3.1 Primary outcome

The primary outcome variable of interest is employment outcome of an adult individual/population, such as status of employment (employed, not employed, etc), type of occupation, and earning (salary, wage or income).

#### 3.3.2 Secondary outcomes

The secondary outcomes are history of welfare dependency, absenteeism and/or presenteeism.

### 3.4 Search methods for identification of studies

We will search electronic databases, grey literature and reference lists of included studies to retrieve studies for the review.

#### 3.4.1 Electronic searches

A mix of health, psychology, sociology and economics databases are selected in relation to the concepts of the literature review. Peer‐reviewed articles will be searched in MEDLINE (EBSCOhost), Psychology and Behavioural Sciences Collection (APA PsycInfo) (EBSCOhost), ECONLIT (Ovid), Sociological Abstracts (ProQuest), Social Science Research Network (SSRN) and Scopus. A search strategy is created in MEDLINE using keywords and Subject Headings related to the concepts of ‘adverse childhood experience’ and ‘employment’ (see Supporting Information S1: Appendix 1). The search strategy will be customised to the rest of the databases. Assistance was sought from a librarian for creating the search strategy. Studies published between 2000 and 2024 will be included because studies assessing the impact of ACEs on various adult outcomes widely proliferated over the past 25 years following the seminal work by Felitti et al. (1998). Studies will be excluded if full text cannot be available either through databases or from authors within 1 month of contact. We will update the search before publication. All searches will adhere to copyright legislations and terms and conditions of use of retrieved and downloaded studies including studies which might be obtained from authors directly.

#### 3.4.2 Searching other resources

In addition to electronic databases, a grey literature search will be conducted on websites of World Health Organisation, OECD, Early Intervention Foundation, and Social Science Research Network (SSRN) site. Further, searching will be done through handsearching of reference lists of included studies and citation tracking (backward and forward citation tracking) in Google Scholar. We will contact authors if we need additional information or if full text cannot be accessed.

#### 3.4.3 Data collection and analysis

##### Description of methods used in primary research

We anticipate the primary method of research for the studies will be cross sectional data analysis using appropriate regression models to assess association between ACEs and employment or differences in outcomes. Studies may also use simple tables to describe relative risk of the outcomes for measured exposure to ACEs.

##### Selection of studies

Studies retrieved from different sources will be collated in EndNote Reference Manager, and duplicates will be removed. The deduplicated studies will be exported to Rayyan Software for screening. Two reviewers will independently screen the selected studies on Rayyan. We will use the ‘blind on’ function to eliminate bias in decision among the reviewers. The ‘blind on’ function in Rayyan does not allow reviewers to see the decisions made by their collaborators. The two reviewers will independently screen the studies for eligibility in two steps: first, screening the titles and abstracts of the studies; second, full‐text screening of studies included in the first screening. Any inconsistencies in decision‐making, between the first and second reviewer will be resolved through discussion with the third reviewer. The review will entirely be conducted manually; no machine learning will be used.

#### 3.4.4 Data extraction and management

The following data will be extracted from the studies that will be included for the synthesis:

- Study characteristics: authors, year, country.
- Methods: study design (observational, cross‐sectional, longitudinal) study setting, data source, sampling, sample size, and method of data analysis.
- Participant characteristics: age, sex, race, ethnicity, and educational status.
- Primary outcome variable: employment outcomes (percentages, odds ratio, difference, standard errors, *p*‐values).
- Secondary outcome variable: welfare dependency, absenteeism, presenteeism.
- Independent variable of interest: type of ACEs and definitions of ACEs.
  ○ Adversity at home.
    ▪ Abuse: emotional, physical, sexual.
    ▪ Neglect: emotional, physical.
    ▪ Household dysfunction: mental illness in the household, domestic violence, divorce/separation of parents, incarcerated parent/relative, and substance abuse by household members.
  ○ Adversity outside the home.
    ▪ Experiencing or witnessing racism, bullying, community violence, living in unsafe neighbourhood and foster care.
- Sources of data on ACEs: retrospective or prospective sources of ACEs include service records, prospective cohort surveys, retrospective cross‐sectional studies, and concurrent cross‐sectional surveys.
- Moderates of effect:
  ○ If studies report any moderators of effect, this will be collected.
  ○ Percentage or mean values for differences explained by moderators of effect.
- Funding sources and declaration of interest of included studies.
- Limitation and notes about of the study.

A data extraction tool, which includes the above listed data, will be developed. The data extraction template will be piloted on five randomly selected studies and will be revised based on feedback form the pilot. Two reviewers will independently extract data using the data extraction template. If there is missing data in the studies, we will contact the authors to fill the gap in the data. We will follow up with the authors weekly three times by sending reminders. If the contacted authors do not respond within 1 month, we will use the available data on the published articles and draw conclusions based on that, and limitations will be noted.

#### 3.4.5 Assessment of risk of bias in included studies

Risk of bias of the studies will be assessed using the NHLBI‐NIH quality assessment tools for observational cohort and cross‐sectional studies (14 items tools) and case‐control studies (12 items tool) (https://www.nhlbi.nih.gov/health-topics/study-quality-assessment-tools). The quality of each study will be rated by one reviewer. A second reviewer which check quality of five randomly selected studies. Differed ratings will be resolved through discussion and risk of bias checked for the rest of the studies.

#### 3.4.6 Unit of analysis issues

We expect the studies that will be included in this review to have individuals as a unit of analysis, given the nature of studies for the review. However, we will cautiously ensure that the unit of analysis is the same across studies. Furthermore, if data permits a meta‐analysis, we will ensure that the same groups will not be included twice in one meta‐analysis.

##### Criteria for determination of independent findings

If there are multiple reports of a single study, we will extract data from all reports. If multiple reports present the same outcome, we will examine sample design (study participants, sample sizes, funding, etc.) to judge whether the different articles are about the same study or not. If there are conflicting finding of the same study by different reports, we will contact the authors for clarification.

##### Dealing with missing data

For a meta‐analysis, where possible, missing data (e.g., relative risk, means, standard deviations) will be computed from available statistics. If not, we will contact the authors of the studies to get the needed information. If we cannot get the missing information, either from the available data or the authors, we will exclude the studies with the missing data from meta‐analyses. Then, we will discuss the issue with the missing data and its potential impact on the result of the review in the discussion section.

##### Assessment of heterogeneity

Heterogeneity will be assessed using forest plot, *Q*‐test, and *I* ^2^ statistics. Initially, we will plot the results of meta‐analysis using forest plot to visually inspect overlaps in the confidence intervals of the studies. Then, we will compute the *Q*‐statistic and *I* ^2^ statistic to statistically evaluate heterogeneity. The Q‐test tests the null hypothesis that the included studies for the meta‐analysis are homogeneous. The *I* ^2^ statistics is computed from Q‐statistics. The value of the *I* ^2^ statistic varies between 0% and 100% with a value closer to 0 indicating non‐heterogeneity. The suggested thresholds for *I* ^2^ test are 25% (low heterogeneity, 50% (moderate heterogeneity) and 75% (high heterogeneity). Interpretation of *Q* and *I* ^2^ statistics will be made cautiously, considering the bias that might arise due to number of included studies (Higgins, 2003).

##### Assessment of reporting biases

Reporting bias will be assessed through fennel plots and Egger's test (Egger et al., 1997; Higgins et al., 2023). We will visually inspect fennel plots to examine asymmetry. This will be complemented by Egger's statistical test of asymmetry.

#### 3.4.7 Data synthesis

Two types of data synthesis will be used: (1) a narrative synthesis and (2) a statistical analysis.

##### Narrative synthesis

Narrative synthesis will be used to synthesise evidence from the included studies using texts and summary table whether we are able to perform pooled statistical analysis or not. The narrative synthesis will explore relationships within and between studies to explain any differences in the relationship between ACEs (type of ACEs, timing of ACEs, number of ACEs experienced) and the primary and secondary outcomes. First, we will present findings of the employment outcomes followed by outcomes of welfare dependency, presenteeism and absenteeism and ACEs. We will present a summary table that describes the characteristics and results of individual studies. We will explore data to see how the authors have controlled for the effect of confounding variables and explain any differences in the results considering differences in the measurement of ACEs, employment outcomes, or characteristics of the respondents. Furthermore, we will summarise mediators of association if the included studies report any.

##### Statistical analysis

The outcome and exposure variable may be measured in various ways, and this may pose a challenge for pooled statistical analysis. However, if data allows appropriate analytical methods will be used to provide pooled synthesis depending on how the outcome variable is measured. Studies will be grouped based on the similarity of measurement of outcomes and ACEs. Random effects meta‐analysis will be conducted for the primary outcome if data allows. In studies that report the employment outcomes as binary outcome variable (e.g., employed, not employed), with ACEs measured as dichotomous variable, pooled odds ratios and 95% confidence intervals will be calculated to reflect the association between employment outcomes and ACEs. In studies that report quantitative employment outcomes (e.g., salary from employment), mean differences in outcomes between the study population with and without ACEs will be calculated. For instance, if we find adequate number of studies reporting employment outcome as dichotomous variable (employed or unemployed) with a dummy exposure variable (ACEs or no ACEs), we will estimate the relative risk to measure association for each study. If studies report adjusted (and unadjusted) measures, analysis will be conducted by stratifying adjusted and unadjusted outcome variable estimates. Furthermore, if feasible, sub‐group analysis will be performed based on the type of ACEs, timing of ACEs, number of ACEs, and characteristics of study subjects such as sex, age groups, and intersection of individual characteristics. There may be differences in measurement of ACEs across studies, including definition of ACEs or categories of ACEs included in the studies. This variation may have an impact on the review results. We will use definitions of ACEs used in the studies and note limitations. Furthermore, methodological limitations of the source of ACE data (e.g., service records, retrospective cross‐sectional studies, or prospective cohort surveys) and impact of the survey designs on the relationship between ACEs and employment outcomes will be discussed. Limitations arising from the inclusion of a selection of ACEs in comparison to other childhood adversities (not included in this synthesis) that impact adult employment outcomes will also be discussed.

#### 3.4.8 Subgroup analysis and investigation of heterogeneity

If at least 10 studies are included in the meta‐analysis and we detect high heterogeneity, we will conduct meta‐regression to explore a potential influence of confounding variables on the outcome. We will include age, gender, socioeconomic status, and study design as confounding variables to investigate heterogeneity.

#### 3.4.9 Sensitivity analysis

Quality of studies may impact robustness of meta‐analysis. To check this, we will perform sensitivity analysis by excluding studies with low quality.

#### 3.4.10 Summary of findings and assessment of the certainty of the evidence

The certainty of the evidence will be evaluated using the Grading of Recommendations Assessment, Development and Evaluation (GRADE) system (Guyatt et al., 2008). Based on the GRADE system, the quality of evidence will be classified as high, moderate, low and very low. The domains for assessing quality of evidence includes risk of bias, inconsistency of results, study limitations, imprecision, indirectness, and publication bias (Guyatt et al., 2008). Summary of the findings including the grading of quality of evidence from GRADE system will be presented in table.

## CONTRIBUTIONS OF AUTHORS

Paul McCrone, PhD is a Professor of Healthcare Economics who has published broadly on international journal on topics of health economics and mental health. Paul has conducted several systematic reviews including the economic impact of mental health. He will contribute to all stages of the review through providing overall guidance on searching, screening, data extraction, and critical review of drafts.

Amarech Obse, PhD, is an experienced health economist who has worked on areas of health financing, equity, inequality, and effects of mental health intervention on inequality. Amarech has training and experience in systematic reviews. She will contribute to database searching, screening, data extraction, writing first draft, and revisions of the manuscript.

Evdoxia Gkaintatzi, is an experienced research fellow in health economics who has worked in building health economic models and conducting scientific and market access‐related research analyses. Evdoxia has working experience in systematic reviews. She will contribute to screening, data extraction, writing first draft, and revisions of the manuscript.

Mina Stoynova, is a librarian and information specialist. She has experience in searching of studies for systematic reviews. She has contributed in creating the database search strategy.

- Content: Paul McCrone, Amarech Obse.
- Systematic review methods: Paul McCrone, Evdoxia Gkaintatzi, Amarech Obse.
- Statistical analysis: Paul McCrone, Evdoxia Gkaintatzi, Amarech Obse.
- Information retrieval: Mina Stoynova, Amarech Obse.

## DECLARATION OF INTEREST

None.

## PRELIMINARY TIMEFRAME

Approximate time for completion of the review is 1 year from the time the protocol is approved.

## PLANS FOR UPDATING THE REVIEW

We will update the search before submitting the manuscript of the review for publication.

## SOURCES OF SUPPORT

UK Research and Innovation [UKRI] award (MR/W002183/1).

## Supporting information

Supporting information.

## Data Availability

N/A
