# Supplementary material for: PROTOCOL: The association between adverse childhood experiences and employment outcomes: Protocol for a systematic review
Source: Campbell Syst Rev. 2024 Oct 31;20(4):e70002. doi: 10.1002/cl2.70002 (PMC11528038; doi:10.1002/cl2.70002)
Supplement: Supplementary file 1 — Supporting information. [file CL2-20-e70002-s001.docx]

**Appendix 1**

**Revised search strategy: Interface - EBSCOhost Research Databases; Search Screen - Advanced Search; Database – MEDLINE**

| **#** | **Query** | **Limiters/Expanders** | **Results** |
| --- | --- | --- | --- |
| S40 | S32 AND S35 | Limiters - Publication Date: 20000101-20241231; Age Related: Adult: 19-44 years, Middle Aged: 45-64 years; Search modes - Proximity | 2,269 |
| S39 | S32 AND S35 | Search modes - Proximity | 6,087 |
| S38 | S33 OR S34 | Search modes - Proximity | 1,240,211 |
| S37 | (MH "Employment+") OR (MH "Income") OR (MH "Remuneration") OR (MH "Employment, Supported") OR (MH "Unemployment") OR (MH "Salaries and Fringe Benefits") | Search modes - Proximity | 148,239 |
| S36 | employ* OR income* OR salary* OR wealth* OR "economic outcome*" OR "labo#r force" OR "human capital" | Search modes - Proximity | 1,194,877 |
| S35 | S1 OR S2 OR S3 OR S4 OR S5 OR S6 OR S7 OR S8 OR S9 OR S10 OR S11 OR S12 OR S13 OR S14 OR S15 OR S16 OR S17 OR S18 OR S19 OR S20 OR S21 OR S22 OR S23 OR S24 OR S25 OR S26 OR S27 OR S28 OR S29 OR S30 OR S31 | Search modes - Proximity | 74,752 |
| 34 | (MM "Bullying+") | Search modes - Proximity | 6,143 |
| 33 | Bully* AND ( (child* OR youth* OR adolescence) n5 adversity ) NOT TI(intervention OR therapy* OR psychotherapy*) | Search modes - Proximity | 134 |
| 32 | Bully* AND ( child* adversity OR adverse child* experience* OR child* trauma OR early life stress OR early life adversity ) | Search modes - Proximity | 442 |
| S31 | unsafe neighbo#rhood* AND ( child* adversity OR adverse child* experience* OR child* trauma OR early life stress OR early life adversity ) | Search modes - Proximity | 14 |
| S30 | unsafe neighbo#rhood* | Search modes - Proximity | 189 |
| S29 | expanded adverse childhood experience | Search modes - Proximity | 10 |
| S28 | ( ( (MH "Exposure to Violence") OR (MH "Violence") ) ) AND ( child* adversity OR adverse child* experience* OR child* trauma OR early life stress OR early life adversity ) | Search modes - Proximity | 769 |
| S27 | residence characteristics AND ( child* adversity OR adverse child* experience* OR child* trauma OR early life stress OR early life adversity ) | Search modes - Proximity | 155 |
| S26 | community violence exposure AND ( child* adversity OR adverse child* experience* OR child* trauma OR early life stress OR early life adversity ) | Search modes - Proximity | 93 |
| S25 | racism AND ( (child* OR youth* OR adolescence) n5 adversity ) NOT TI(intervention OR therapy* OR psychotherapy*) | Search modes - Proximity | 38 |
| S24 | racism AND (adverse childhood experiences) | Search modes - Proximity | 84 |
| S23 | racism AND ( (child* OR youth* OR adolescence) n5 adversity ) | Search modes - Proximity | 38 |
| S22 | (MM "Foster Home Care") AND ( child* adversity OR adverse child* experience* OR child* trauma OR early life stress OR early life adversity ) | Search modes - Proximity | 116 |
| S21 | "foster care" AND ( child* adversity OR adverse child* experience* OR child* trauma OR early life stress OR early life adversity ) | Search modes - Proximity | 247 |
| S20 | (MM "Parental Death+") AND ( child* adversity OR adverse child* experience* OR child* trauma OR early life stress OR early life adversity ) | Search modes - Proximity | 28 |
| S19 | ( ( (parent* OR mother* OR father* ) n5 death ) ) AND ( child* adversity OR adverse child* experience* OR child* trauma OR early life stress OR early life adversity ) | Search modes - Proximity | 264 |
| S18 | ( ( (parent* OR mother* OR father* OR relative*) n5 incarcerat* ) ) AND ( child* adversity OR adverse child* experience* OR child* trauma OR early life stress OR early life adversity ) | Search modes - Proximity | 126 |
| S17 | separat* AND ( child* adversity OR adverse child* experience* OR child* trauma OR early life stress OR early life adversity ) | Search modes - Proximity | 2,190 |
| S16 | divorce AND ( child* adversity OR adverse child* experience* OR child* trauma OR early life stress OR early life adversity ) | Search modes - Proximity | 359 |
| S15 | ( ( (parent* OR mother* OR father*) n5 alcohol* ) AND ( child* adversity OR adverse child* experience* OR child* trauma OR early life stress OR early life adversity ) | Search modes - Proximity | 164 |
| S14 | "domestic violence" AND ( child* adversity OR adverse child* experience* OR child* trauma OR early life stress OR early life adversity ) | Search modes - Proximity | 669 |
| S13 | (MH "Child of Impaired Parents") AND (substance abuse OR substance abuse disorder* OR substance use OR drug abuse OR drug addiction OR drug use) | Search modes - Proximity | 848 |
| S12 | (MH "Child of Impaired Parents") AND ( mental health OR mental illness OR mental disorder OR psychiatric illness ) | Search modes - Proximity | 1,795 |
| S11 | "child of impaired parent*" AND (substance abuse OR substance abuse disorder* OR substance use OR drug abuse OR drug addiction OR drug use) | Search modes - Proximity | 850 |
| S10 | "child of impaired parent*" AND ( mental health OR mental illness OR mental disorder OR psychiatric illness ) | Search modes - Proximity | 1,802 |
| S9 | ( (parent* OR mother* OR father* OR relative*) n5 substance abuse ) AND ( child* adversity OR adverse child* experience* OR child* trauma OR early life stress OR early life adversity ) | Search modes - Proximity | 131 |
| S8 | ( ( (parent* OR mother* OR father* OR relative*) n5 "mental illness" ) AND ( child* adversity OR adverse child* experience* OR child* trauma OR early life stress OR early life adversity ) | Search modes - Proximity | 163 |
| S7 | household dysfunction AND ( child* adversity OR adverse child* experience* OR child* trauma OR early life stress OR early life adversity ) | Search modes - Proximity | 326 |
| S6 | ( "child* neglect" OR "physical neglect" OR "emotional neglect" ) | Search modes - Proximity | 2,836 |
| S5 | ( "child* abuse" OR "physical abuse" OR "emotional abuse" OR "sexual abuse" ) | Search modes - Proximity | 53,447 |
| S4 | (SU "Adverse Childhood Experiences") OR (SU "Child Abuse+") OR (SU "Child Abuse, Sexual") | Search modes - Proximity | 16,553 |
| S3 | (MM "Child Abuse+") OR (MM "Child Abuse, Sexual") | Search modes - Proximity | 28,845 |
| S2 | (MM "Adverse Childhood Experiences") | Search modes - Proximity | 3,731 |
| S1 | TI ( "adverse child* experience*" OR "traumatic child* experience*" OR "child* adversity" OR "child* trauma" OR "child* maltreatment" OR "child* mistreatment" OR "early life stress" OR "early life trauma" OR "early life adversity") OR AB( "adverse child* experience*" OR "traumatic child* experience*" OR "child* adversity" OR "child* trauma" OR "child* maltreatment" OR "child* mistreatment" OR "early life stress" OR "early life trauma" OR "early life adversity") | Search modes - Proximity | 21,472 |
